# Tuning the Wettability and Surface Free Energy of Poly(vinylphenol) Thin Films by Modulating Hydrogen-Bonding Interactions

**DOI:** 10.3390/polym12030523

**Published:** 2020-03-01

**Authors:** Chih-Feng Wang, Dula Daksa Ejeta, Jian-Yi Wu, Shiao-Wei Kuo, Ching-Hsuan Lin, Juin-Yih Lai

**Affiliations:** 1 Advanced Membrane Materials Research Center, Graduate Institute of Applied Science and Technology, National Taiwan University of Science and Technology, Taipei 106, Taiwan; duladaksa@gmail.com (D.D.E.); jylai@mail.ntust.edu.tw (J.-Y.L.); 2 Department of Materials Science and Engineering, I-Shou University, Kaohsiung 840, Taiwan; jam-e@yahoo.com.tw; 3 Department of Materials and Optoelectronic Science, Center of Crystal Research, National Sun Yat-Sen University, Kaohsiung 804, Taiwan; 4 Department of Medicinal and Applied Chemistry, Kaohsiung Medical University, Kaohsiung 807, Taiwan; 5 Department of Chemical Engineering, National Chung Hsing University, Taichung 402, Taiwan; linch@dragon.nchu.edu.tw; 6 R&D Centre for Membrane Technology, Chung Yuan University, Taoyuan 320, Taiwan

**Keywords:** poly(vinyl phenol), surface free energy, intermolecular interaction, hydrogen bonding

## Abstract

The ability to tune the surface properties of a polymer film in a simple and effective manner is important for diverse biological, industrial, and environmental applications. In this work, we investigated whether or not the surface free energy of poly(vinyl phenol; PVPh) can be tuned by adjusting the casting solvent and the thermal treatment time, which alters the proportions of intra-and intermolecular hydrogen bonding interactions. Compared to the untreated sample, in tetrahydrofuran (THF) system, the thermal treatment resulted in a lower proportion of intermolecular hydrogen bonds and a concomitant decrease in the surface free energy (from 39.3 to 18.8 mJ/m^2^). In contrast, the thermal treatment in propylene glycol methyl ether acetate (PGMEA) and ethyl-3-ethoxypropionate (EEP) systems increased the proportion of intermolecular hydrogen bonds and the surface free energy of the polymer thin films, from 45.0 to 54.3 mJ/m^2^ for PGMEA and from 45.5 to 52.9 mJ/m^2^ for EEP. Controlling intermolecular hydrogen-bonding interactions is a unique and easy method for tuning the surface free energies of polymer substances.

## 1. Introduction

The surface properties of a solid substance, such as adhesion, wettability, and surface roughness are significant characteristics for biological, industrial, and environmental applications. Current approaches for shaping the wettability properties and surface free energy of a polymer material include plasma modification or other methods [1,2,3,4]. Intra- and intermolecular hydrogen bonds play significant roles in defining the surface properties of a polymeric material. Hydrogen bonds can be intramolecular (within a single molecule) or intermolecular (between molecules). An intramolecular hydrogen bond is formed when both the hydrogen bond donor and hydrogen bond accepter exist within the same molecule, whilst an intermolecular hydrogen bond is formed between two different molecules. In a given polymer chain, either intra- or inter-chain hydrogen bonding can be built by the same functional groups [5]. For instance, polypeptide α-helix and β-sheet structures are stabilized by inter-chain and intra-chain hydrogen bonds, respectively [6,7].

Jiang et al. [8] revealed that intramolecular hydrogen bonding between the N–H and C=O groups of poly(*N*-isopropylacrylamide; PNIPAAm) main chains induces low surface free energies and a high contact angle of water at temperatures above the lower critical solution temperature (LCST). In contrast, intermolecular hydrogen bonding between water and the PNIPAAm chains results in higher surface free energy and a lower contact angle of water at temperatures below the LCST. Furthermore, Chung and his coworker found that the presence of amide groups in main-chain-fluorinated liquid crystalline polymers enable strong intermolecular hydrogen bonding that result in enhanced surface free energies and lower hydrophobicities [9]. We have also previously reported that as-prepared polybenzoxazines have surface free energies as low as 16.4 mJ/m^2^, which is even lower than that of polytetrafluoroethene (PTFE; 21 mJ/m^2^), a result of strong intramolecular hydrogen bonding [10]. Nevertheless, increasing the number of intermolecular hydrogen bonds between –OH groups raises the surface free energies of polybenzoxazines. We have shown that a portion of the intramolecular hydrogen bonds in the polybenzoxazine system converts into intermolecular hydrogen bonds upon exposure to UV radiation, resulting in a higher surface free energy and, consequently, a higher hydrophilicity [11,12]. Lin et al. found that as-cured main-chain-type polybenzoxazine precursors also exhibit strong intramolecular hydrogen bonding and low surface free energies [13,14]. Apart from the polybenzoxazine system, we have also revealed that poly(vinylphenol; PVPh) exhibits a tremendously low surface energy through thermal treatment due to a loss in the fraction of intermolecular hydrogen bonds involving its –OH groups [15,16]. Xin et al. reported that the condensation reactions of polysiloxanes lead to a reduction in the number of intramolecular hydrogen bonds, resulting in an increase in surface free energy and hydrophilicity [17]. Furthermore, the strengths of the intra- or intermolecular hydrogen bonds and the nanometric polymer thin-film structure strongly depend on the type of solvent used in the coating process.

In the current work, we prepared three PVPh thin films by dissolving PVPh in three different solvents and then spin coated the solutions onto glass slides. Relationships between the surface free energies of the PVPh thin films and the hydrogen bond strengths before and after heat treatment were systematically and comprehensively studied by Fourier-transform infrared (FT-IR) spectroscopy and contact angle measurements. We discovered that the surface free energy of a PVPh thin film is tunable over the 18.8–54.3 mJ/m^2^ range by merely changing the casting solvent and the thermal treatment time.

## 2. Experimental Procedure

The PVPh (Mw: 11,000 g/mol) used in this study was supplied by Sigma–Aldrich, St. Louis, MA, USA.Polymer stock solutions were prepared by dissolving PVPh in tetrahydrofuran (THF), propylene glycol methyl ether acetate (PGMEA), and ethyl-3-ethoxypropionate (EEP) at concentrations of 30 mg/mL. Each solution was filtered through a PTFE syringe filter (0.2 μm), after which 1.5 mL of the polymer solution was spin-coated onto a glass slide (1 mm × 50 mm × 50 mm) using a photoresist spinner (1500 rpm, 45 s). The spin-coated sample was left to dry for 2 h at 65 °C. The as-prepared polymer film was thermally treated by placing it in an oven at 180 °C for the desired time. PVPh films prepared from THF, PGMEA, and EEP are referred to as “PVPh-T”, “PVPh-P”, and “PVPh-E”, respectively.

FT-IR spectra of the PVPh polymer films were acquired using the potassium bromide (KBr) plate method. The sample was prepared by casting the solution directly onto a KBr plate followed by treatment under conditions similar to those used for bulk preparation. FT-IR spectra were recorded on a Nicolet Avatar 320 FT-IR spectrophotometer (Thermo Fisher, Waltham, MA, USA) with 32 scans and a spectral resolution of 1 cm^−1^. The advancing-liquid contact angle on each polymer film was measured through a FDSAMagicDroplet-100 contact angle goniometer by injecting a 5 μL liquid droplet. Surface free energies were measured using diiodomethane (DIM; 99%; Sigma–Aldrich) and deionized water as standards. Surface roughness profiles of the polymer film structures were determined by atomic force microscopy (AFM) using a Digital Instruments DI5000 scanning probe microscope. The values of rootmeansquare (rms) roughness were calculated over scan areas 5 μm × 5 μm in size. UV-vis spectrum was performed by Thermo Scientific Evolution-201 UV-vis spectrophotometer.

## 3. Results and Discussion

PVPh exhibits good characteristics for the formation of spin-coated films on glass slides and was used as the target polymer in this study. The PVPh films prepared through THF, PGMEA, and EEP were abbreviated as “PVPh-T”, “PVPh-P”, and “PVPh-E”, respectively. The chemical structures of PVPh, THF, PGMEA, and EEP are showed in Figure S1. Temperatures above the glass-transition temperature (*T*g) of a polymer are well-known to disrupt hydrogen bonds. These bonds reform in a different (inter- and intramolecular) distribution relative to that prior to thermal annealing upon fast cooling to ambient temperature, which is why we chose 180 °C as the thermal treatment temperature.

The surface free energy of a solid can determine its adhesive properties; however, determining the solid surface free energy directly is arduous, and procedures based on contact angle measurements are commonly employed for practical reasons [18]. Since the advancing contact angle is less sensitive to heterogeneity and surface roughness, the components of surface and interfacial tension are usually calculated using advancing-angle data. Surface free energies were evaluated using the two-liquid geometric method [19], in which the solid surface free energy ($\gamma_{S}$) is determinedby the conjunction of twocomponents (Equation (1)):

(1) $$\gamma_{S}=\gamma_{S}^{d}+\gamma_{S}^{p}$$

where $\gamma_{S}^{d}$ is the dispersion component and $\gamma_{S}^{p}$ is the polar component $\gamma_{S}^{d}$ and $\gamma_{S}^{p}$ of a solid can be evaluated using Equation (2) after measuring the contact angles (θ) of two characteristic liquids:

(2) $$\begin{array}{c} \gamma_{L1}\left({1+\cos\theta }_{1}\right)=2{\left(\gamma_{S}^{d}\gamma_{L1}^{d}\right)}^{\frac{1}{2}}+2{\left(\gamma_{S}^{p}\gamma_{L1}^{p}\right)}^{\frac{1}{2}}\\ \gamma_{L2}\left(1+{\cos\theta }_{2}\right)=2{\left(\gamma_{S}^{d}\gamma_{L2}^{d}\right)}^{1/2}+2{\left(\gamma_{S}^{p}\gamma_{L2}^{p}\right)}^{1/2} \end{array}$$

where $\gamma_{L}$ is the surface tension of the liquid.

The advancing contact angles (ACAs) of DIM and water on PVPh-T increased with increasing treatment time, from an initial value of 44° to 78° after 8 h in the case of DIM, and from 76° to 101° after 8 h for water (Table 1). These data reveal that the total surface free energy declined considerably, from 39.3 to 18.8 mJ/m^2^, as the treatment time was increased from 0 to 8 h, in good agreement with our previous results [15]. However, PVPh-P and PVPh-E exhibited completely different results. The ACA of DIM increased slightly with increasing treatment time for PVPh-P, from an initial value of 42° to 47° after 8 h (Table 2). In contrast, the ACA of water was observed to decrease, from 63° after 0 h to 45° after 8 h. These results reveal that the total surface free energy increased substantially, from 45.0 to 54.3 mJ/m^2^, as the treatment time was increased from 0 to 8 h. The ACAs of DIM and water for PVPh-E followed similar trends to those observed for PVPh-P (Table 3); the ACAs of DIM slightly increased with increasing treatment time (from 42° after 0 h to 48° after 8 h), while that of water decreased with increase treatment time(from 62° after 0 h to 47° after 8 h), which means that the total surface free energy increased substantially from 45.5 to 52.9 mJ/m^2^ as the treatment time was increased from 0 to 8 h. We characterized the PVPh polymer film in terms of its FT-IR spectra to understand the mechanisms behind the surface free energy changes.

**Figure 1:**
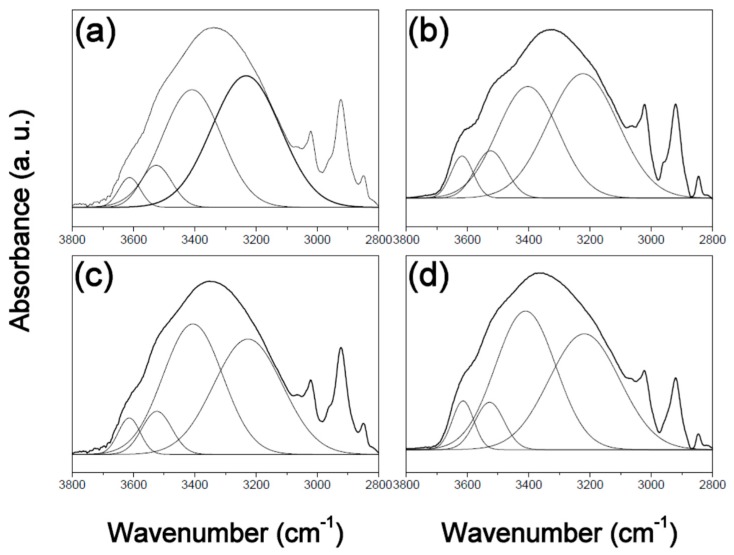
Fourier-transforminfrared (FT-IR)spectra of poly(vinyl phenol) prepared in tetrahydrofuran(PVPh-T) thermally treated at 180 °C for (**a**) 0, (**b**) 1, (**c**) 4, and (**d**) 8 h, overlaid with Gaussian-fitted curves.

**Figure 2:**
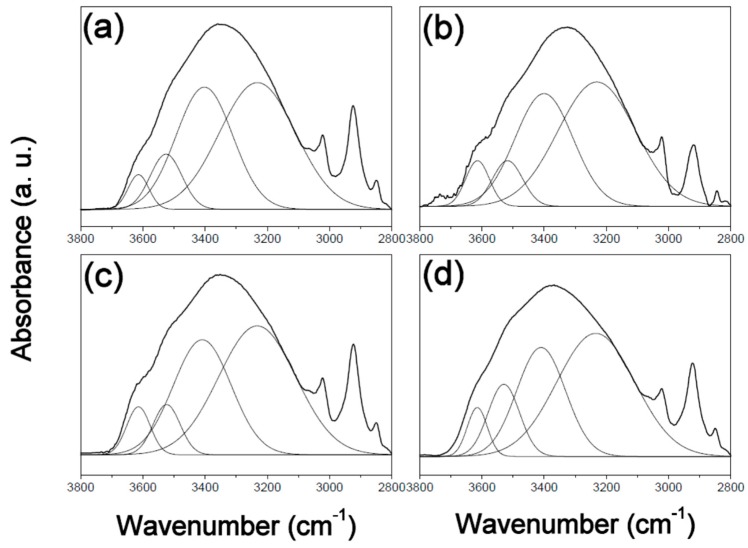
FT-IR spectra of poly(vinyl phenol) prepared in propylene glycol methyl ether acetate (PVPh-P) thermally treated at 180 °C for (**a**) 0, (**b**) 1, (**c**) 4, and (**d**) 8 h, overlaid with Gaussian-fitted curves.

**Figure 3:**
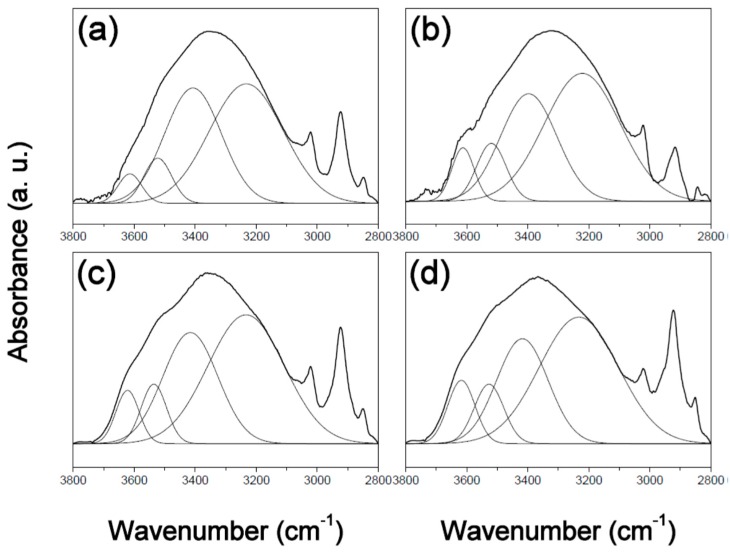
FT-IR spectra of poly(vinyl phenol) prepared in ethyl-3-ethoxypropionate (PVPh-E) thermally treated at 180 °C for (**a**) 0, (**b**) 1, (**c**) 4, and (**d**) 8 h, overlaid with Gaussian-fitted curves.

In order to understand the mechanism responsible for the observed changes in surface free energy, the PVPh polymer films in this study were characterized by FT-IR spectroscopy. Figure 1, Figure 2 and Figure 3 display expanded FTIR spectra in the 3800–2800 cm^−1^ region for the three films, before and after thermal treatment at 180 °C for different times. The spectrum of the PVPh polymer exhibits a hydroxyl absorbance band that can be fitted by four Gaussian functions, namely a narrow shoulder absorbance band at 3650–3580 cm^−1^ that corresponds to free hydroxyl groups, an absorbance peak at 3550–3515 cm^−1^ that corresponds to weak hydroxyl-π hydrogen bonding, an absorbance peak at 3450–3350 cm^−1^ that corresponds to hydrogen-bonded hydroxyl groups in linear chains of intramolecular hydrogen bonding, and one at 3234–3110 cm^−1^ due to hydroxyl groups involved in hydrogen-bonded “dimers” (OH–OH) and multiple hydrogen-bonding interactions in cyclic structures (intermolecular hydrogen bonding) [20,21].

**Figure 4:**
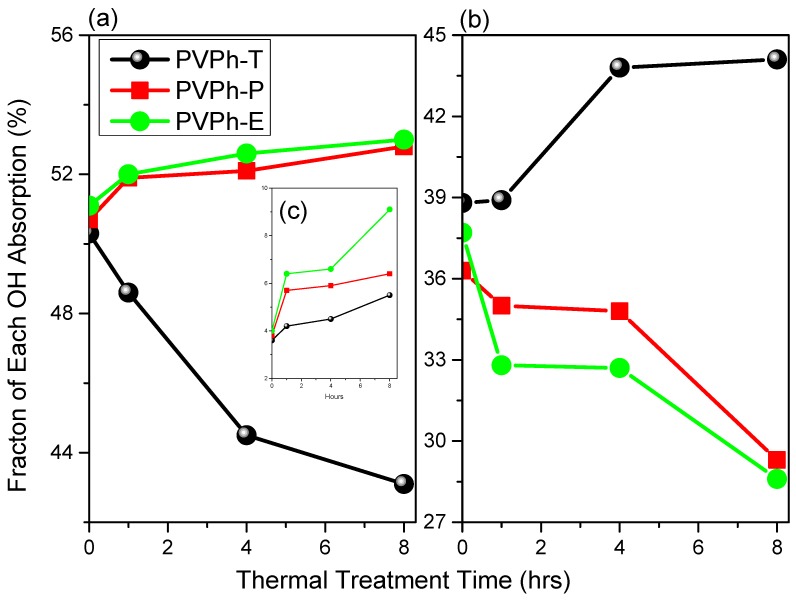
Fractions of (**a**) intermolecular hydrogen bonds, (**b**) intramolecular hydrogen bonding, and (**c**) free OH in PVPh-T, PVPh-P, and PVPh-E thermally treated for different times.

All curing results are summarized in Tables S1–S3 and Figure 4 by using Gaussian function by controlling the wavenumber and half-width within ± 10 cm^−1^. In our previous report, we revealed that the free energies of polymer surfaces can be altered by changing the degree of intra- or intermolecular hydrogen bonding [10,11,12,13,14,15,16]. A decrease in intermolecular hydrogen bonding leads to a decrease in the surface free energy of a polymer; conversely, the surface free energy of a polymer increases with increasing intermolecular hydrogen bonding. As mentioned above, heat treatment at high temperatures generally disrupts hydrogen bonding, and differently distributed hydrogen bonds (intra- and intermolecular hydrogen bonded) are formed after rapid cooling to ambient temperature. Firstly, we observed that the area fraction of free hydroxyl was increased with the increase of thermal treatment time as expected in Figure 4c. Secondly, it is clear that the fraction of intermolecular hydrogen bonds in PVPh-T decreased from 50.3% to 43.1% as the thermal treatment time was increased from 0 to 8 h (Figure 4a); this decrease resulted in the thermally treated PVPh-T exhibiting a lower surface free energy. On the contrary, the fraction of intermolecular hydrogen bonds in PVPh-P increased from 50.7% to 52.8% with increasing thermal treatment time (0–8 h; Figure 4a); this increase resulted in the thermally treated PVPh-P having a higher surface free energy. The hydrogen-bonding distribution in PVPh-E followed a similar trend to that observed for PVPh-P; intermolecular hydrogen bonding increased from 51.1% to 53.0% as the thermal treatment time increased from 0 to 8 h (Figure 4a). Furthermore, Figure 4b shows the area fraction of intramolecular hydrogen bonding of PVPh-T, PVPh-P, and PVPh-E, and it shows the completely different trend with the area intermolecular hydrogen bonding. We found that the fraction of intramolecular hydrogen bonding in PVPh-T increased from 38.8% to 44.1%, whereas PVPh-P and PVPh-E both decreased from 36.3% to 29.3% and 37.7% to 28.6%, respectively.

**Figure 5:**
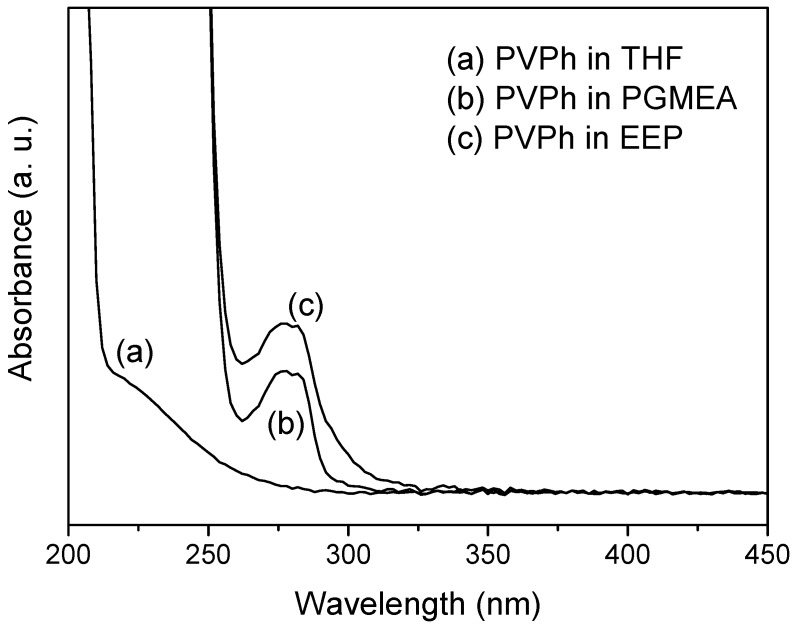
UV-visible absorption spectra of PVPh in various organic solvents: (**a**) tetrahydrofuran (THF), (**b**) propylene glycol methyl ether acetate (PGMEA), and (**c**) ethyl-3-ethoxypropionate (EEP).

The strengths of the intra- or and intermolecular hydrogen bonds and the nanometric structures of the polymer thin films are also strongly dependent on what kinds of solvents are used during the coating process as discussed in detail in our previous studies [7,22,23,24,25,26,27]. For example, in PVPh blending with P4VP, we found that this mixture in good solvent displays separation coil behavior; but it displays complex aggregation in poor solvent [7,22,23]. Similar behavior was also found in PTyr/P4VP blend and complex system [24,25,26]. Polymers appear distended and occupy large volumes a with separated coil in good solvents. Under these conditions, the intermolecular forces between the solvent and the functional groups on the polymer chain dominate over intermolecular interactions. On the other hand, intramolecular forces dominate and chains contract with aggregation in a poor solvent. The interactions between PVPh and the solvents were further investigated by UV-vis spectroscopy (200–450 nm), with the spectra shown in Figure 5. The presence of bands at $\lambda_{\max}$ values of ~276 and ~279 nm in the spectra in PGMEA and EEP, respectively, are assigned to PVPh/solvent complexes formed through intermolecular hydrogen bonding. Shifts in the positions of the absorbance bands are attributed to changes in hydrogen-bonding strengths that are governed by proton-donor and proton-acceptor interactions between the solute and solvent molecules. As shown in Figure 5, the red shifts observed in PGMEA and EEP were larger than those observed in THF, confirming that PVPh interacts more strongly with PGMEA and EEP [28]. Hence, PVPh-T exhibited a relatively low fraction of intermolecular hydrogen bonds (50.3%) compared to those of PVPh-P (50.7%) and PVPh-E (51.1%). The solvent used for PVPh thin film preparation also affects the value of $\gamma_{S}^{p}$ (the polar component of the surface free energy, which includes dipole–dipole, dipole-induced-dipole, and hydrogen-bonding interactions, etc.) of the as-prepared PVPh film. PVPh-T had a relatively low $\gamma_{S}^{p}$ (6.8 mJ/m^2^) compared to those of PVPh-P (14.4 mJ/m^2^) and PVPh-E (15.1 mJ/m^2^). Thermal treatment changed the fractions of intermolecular hydrogen bonds and the $\gamma_{S}^{p}$ values of the PVPh-T, PVPh-P, and PVPh-E systems. After thermal treatment at 180 °C for 8 h, PVPh-T exhibited a 43.1% fraction of intermolecular hydrogen bonds and a $\gamma_{S}^{p}$ of 2.1 mJ/m^2^. On the other hand, the fractions of intermolecular hydrogen bonds in PVPh-P and PVPh-E were higher, at 52.8% and 53.0%, respectively, following thermal treatment (180 °C for 8 h), while the $\gamma_{S}^{p}$ values of PVPh-P and PVPh-E were higher, at 29.9 and 28.7 mJ/m^2^, respectively.

## 4. Conclusions

The surface properties of a polymeric material can be adjusted by tuning the fractions of inter- and intramolecular hydrogen bonds in the polymer. Herein, we adjusted the surface free energies of a PVPh thin film by changing the casting solvent and the thermal-treatment time. A decrease in the fraction of intermolecular hydrogen bonds between the hydroxyl groups in PVPh lead to a decrease in the surface free energy of the polymer thin film. As the result, PVPh-T exhibited the lowest surface free energy (18.8 mJ/m^2^). On the other hand, an increase in the degree of intermolecular hydrogen bonding enhanced the surface free energy, with PVPh-G and PVPh-E exhibiting values of 54.3 and 52.9 mJ/m^2^, respectively. Our study provides interesting guidance for tuning the surface free energies of polymer materials.

## Figures and Tables

**Table 1:** Root-mean-square surface roughness, advancing contact angles for water and diiodomethane, and surface free energies of poly(vinyl phenol) prepared in tetrahydrofuran (PVPH-T).

Temp. (°C)	Time (h)	rms (nm)	Contact Angle (°)		Surface Free Energy (mJ/m^2^)		
			H_2_O	Diiodomethane (DIM)	$\gamma_{S}^{d}$	$\gamma_{S}^{p}$	$\gamma_{S}$
60	2	10.3	76	44	32.5	6.8	39.3
180	1	4.5	90	57	27.8	2.7	30.5
	4	3.3	98	74	18.5	2.5	21.1
	8	7.8	101	78	16.7	2.1	18.8

**Table 2:** Root-mean-square surface roughness, advancing contact angles for water and diiodomethane, and surface free energies of poly(vinyl phenol) prepared in propylene glycol methyl ether acetate (PVPH-P).

Temp. (°C)	Time (h)	rms (nm)	Contact Angle (°)		Surface Free Energy (mJ/m^2^)		
			H_2_O	DIM	$\gamma_{S}^{d}$	$\gamma_{S}^{p}$	$\gamma_{S}$
60	2	1.1	63	42	30.6	14.4	45.0
180	1	4.2	59	46	27.5	18.4	45.9
	4	3.0	49	48	24.5	27.1	51.6
	8	5.6	45	47	24.4	29.9	54.3

**Table 3:** Root-mean-square surface roughness, advancing contact angles for water and diiodomethane, and surface free energies of poly(vinyl phenol) prepared in ethyl-3-ethoxypropionate (PVPH-E).

Temp. (°C)	Time (h)	rms (nm)	Contact Angle (°)		Surface Free Energy (mJ/m^2^)		
			H_2_O	DIM	$\gamma_{S}^{d}$	$\gamma_{S}^{p}$	$\gamma_{S}$
60	2	1.3	62	42	30.4	15.1	45.5
180	1	3.1	57	43	27.7	19.6	47.3
	4	2.4	51	46	26.0	24.7	50.7
	8	2.3	47	48	24.2	28.7	52.9

## Supplementary Materials

The following are available online at https://www.mdpi.com/2073-4360/12/3/523/s1, Figure S1: The chemical structures of PVPh, THF, PGMEA, and EEP, Table S1: FTIR spectrum fitting results of PVPh-T films, Table S2: FTIR spectrum fitting results of PVPh-P films, Table S3: FTIR spectrum fitting results of PVPh-E films.
