# Supplementary material for: Tuning the Wettability and Surface Free Energy of Poly(vinylphenol) Thin Films by Modulating Hydrogen-Bonding Interactions
Source: Polymers (Basel). 2020 Mar 1;12(3):523. doi: 10.3390/polym12030523 (PMC7182822; doi:10.3390/polym12030523)
Supplement: Supplementary file 1 [file polymers-12-00523-s001.pdf]

Supplementary material

Tuning the Wettability and Surface Free Energy of Poly(vinyl phenol) Thin Films by Modulating Hydrogen-Bonding Interactions

Chih-Feng Wang[1]*, Dula Daksa Ejeta[1], Jian-Yi Wu[2], Shiao-Wei Kuo[3, 4]*, Ching-Hsuan Lin[5], Juin-Yih Lai[1, 6]

[1]Advanced Membrane Materials Research Center, Graduate Institute of Applied Science and Technology, National Taiwan University of Science and Technology, Taipei, 106, Taiwan

[2]Department of Materials Science and Engineering, I-Shou University, Kaohsiung, 840, Taiwan.

[3]Department of Materials and Optoelectronic Science, National Sun Yat-Sen University, Kaohsiung, 804, Taiwan.

[4]Department of Medicinal and Applied Chemistry, Kaohsiung Medical University, Kaohsiung, 807, Taiwan

[5]Department of Chemical Engineering, National Chung Hsing University, Taichung 402, Taiwan

[6]R&D Centre for Membrane Technology, Chung Yuan University, Taoyuan, 320, Taiwan

*To whom all correspondence should be addressed

E-mail: cfwang@mail.ntust.edu.tw

kuosw@faculty.nsysu.edu.tw

Tel: 886-2-27301210 (C.-F.W.) 886-525-2000 (S.-W.K.)

Fax: 886-2-27303733(C.-F.W.) 886-525-4099 (S.-W.K.)

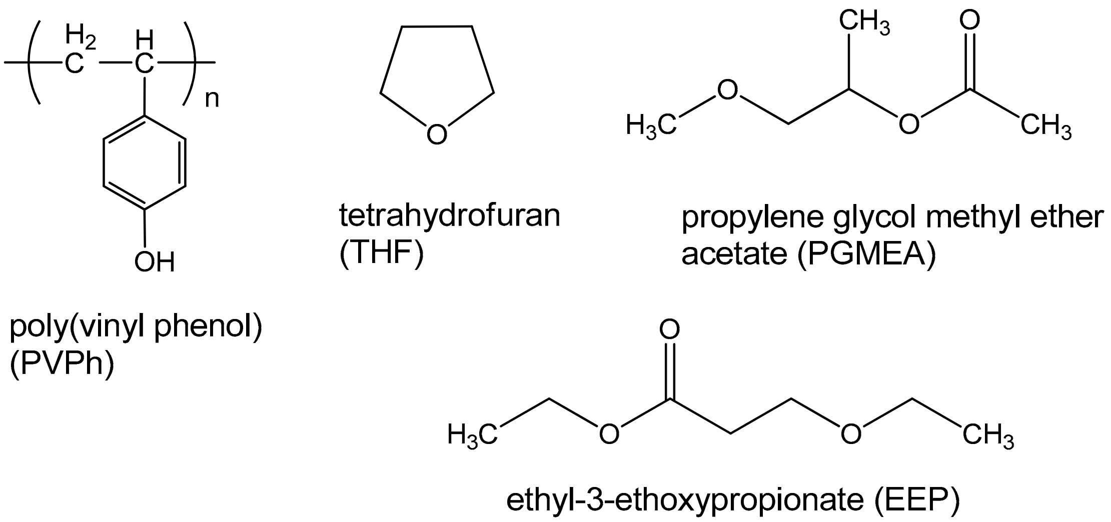

Figure S1. The chemical structures of PVPh, THF, PGMEA, and EEP.

Table S1. FTIR spectrum fitting results of PVPh-T films.

Thermal treatment time (h)		Peak 1 3234–3110 cm^{-1}	Peak 2 3450–3350 cm^{-1}	Peak 3 3550–3515 cm^{-1}	Peak 4 3650–3580 cm^{-1}
0	Position (cm^{-1})	3232	3409	3526	3612
	FWHM (cm^{-1})	264	227	120	82
	Fraction (%)	50.3	38.8	7.3	3.6
1	Position (cm^{-1})	3222	3402	3524	3617
	FWHM (cm^{-1})	269	233	120	85
	Fraction (%)	48.6	38.9	8.3	4.2
4	Position (cm^{-1})	3226	3406	3523	3614
	FWHM (cm^{-1})	263	229	114	84
	Fraction (%)	44.5	43.8	7.2	4.5
8	Position (cm^{-1})	3218	3410	3527	3614
	FWHM (cm^{-1})	271	231	111	83
	Fraction (%)	43.1	44.1	7.3	5.5

Table S2. FTIR spectrum fitting results of PVPh-P films.

Thermal treatment time (h)		Peak 1 3234–3110 cm^{-1}	Peak 2 3450–3350 cm^{-1}	Peak 3 3550–3515 cm^{-1}	Peak 4 3650–3580 cm^{-1}
0	Position (cm^{-1})	3231	3402	3525	3613
	FWHM (cm^{-1})	287	213	120	79
	Fraction (%)	50.7	36.3	9.2	3.8
1	Position (cm^{-1})	3230	3400	3517	3614
	FWHM (cm^{-1})	294	218	114	89
	Fraction (%)	51.9	35.0	7.4	5.7
4	Position (cm^{-1})	3232	3409	3524	3614
	FWHM (cm^{-1})	291	218	102	89
	Fraction (%)	52.1	34.8	7.2	5.9
8	Position (cm^{-1})	3233	3410	3529	3615
	FWHM (cm^{-1})	294	184	119	76
	Fraction (%)	52.8	29.3	11.5	6.4

Table S3. FTIR spectrum fitting results of PVPh-E films.

Thermal treatment time (h)		Peak 1 3234–3110 cm^{-1}	Peak 2 3450–3350 cm^{-1}	Peak 3 3550–3515 cm^{-1}	Peak 4 3650–3580 cm^{-1}
0	Position (cm^{-1})	3232	3407	3522	3614
	FWHM (cm^{-1})	286	219	107	91
	Fraction (%)	51.1	37.7	7.2	4.0
1	Position (cm^{-1})	3222	3396	3519	3612
	FWHM (cm^{-1})	292	217	113	86
	Fraction (%)	52.0	32.8	8.8	6.4
4	Position (cm^{-1})	3233	3415	3536	3621
	FWHM (cm^{-1})	294	212	98	89
	Fraction (%)	52.6	32.7	8.1	6.6
8	Position (cm^{-1})	3232	3417	3527	3617
	FWHM (cm^{-1})	304	199	113	104
	Fraction (%)	53.0	28.6	9.3	9.1
